# Introduction

**DOI:** 10.1007/978-3-030-58948-6_1

**Published:** 2021-03-12

**Authors:** Jungwoo Ryoo, Kurt Winkelmann

**Affiliations:** 1 Pennsylvania State University, Altoona, PA USA; 2 Valdosta State University, Valdosta, GA USA; 3 The Pennsylvania State University, Altoona, PA USA; 4 Valdosta State University, Valdosta, GA USA

## Abstract

The practice of educating students in college-level science, technology, engineering, and math (STEM) subjects is influenced by many factors, including education research, governmental and school policies, financial considerations, technology limitations, and acceptance of innovations by faculty and students. Working together, stakeholders in STEM higher education must find creative ways to address the increasing need for a diverse US workforce with a strong STEM background (President’s Council of Advisors on Science and Technology 2012) and the need for a more STEM-literate general population (National Research Council 2012).

## X-FILEs Approach

The practice of educating students in college-level science, technology, engineering, and math (STEM) subjects is influenced by many factors, including education research, governmental and school policies, financial considerations, technology limitations, and acceptance of innovations by faculty and students. Working together, stakeholders in STEM higher education must find creative ways to address the increasing need for a diverse US workforce with a strong STEM background (President’s Council of Advisors on Science and Technology 2012) and the need for a more STEM-literate general population (National Research Council 2012).

In order to help researchers, developers, educators, and other stakeholders find these creative solutions, we conducted the eXploring the Future of Innovative Learning Environments (X-FILEs) project: a series of agenda-setting, interactive, online activities followed by a face-to-face workshop and a writing initiative which led to this book. Our participants considered the following question:

*What are the near-term and longer-term impacts, opportunities, challenges, and future research initiatives related to the development and implementation of innovative learning environments (ILEs) in higher education STEM disciplines?*

This project included three components: (1) Interactive, online discussions prior to the workshop introduced participants to various innovative learning environments (ILEs) and solicited feedback from a wide range of stakeholders. These discussions formed the basis of the workshop agenda. (2) Participants at our 2-day, face-to-face workshop engaged in creative activities that helped them envision how ILEs can transform STEM higher education. (3) Project leaders (editors of this book) and invited writers continued to synthesize the ideas, questions, challenges, and solutions proposed during the workshop into this book.

The workshop participants discussed and explored four main ILE categories: personalized and adaptive learning, multimodal learning, cross reality (XR), and artificial intelligence (AI) and machine learning (ML). While a particular technology application may fall within one or more of these four categories listed in Table 1, each category is broader than any single educational innovation. Therefore, the usefulness and relevance of our book will endure beyond the shelf life of any current technology application.

**Table 1:** ILE categories and sample technologies and pedagogies

ILE category	Sample technologies and pedagogies of ILEs
Personalized and adaptive learning	Micro-credentialing (badging), self-regulated learning, individualized learning paths, learning analytics, mastery learning, intelligent tutors, student-centered learning
Multimodal learning formats	Digital storytelling, online and blended learning, flipped classroom, game-based learning (GBL), mobile learning, digital publishing, community engagement
Extended/cross reality (XR)	Virtual reality (VR), augmented reality (AR), mixed reality (MR), virtual worlds (VW)
Artificial intelligence (AI) and machine learning (ML)	Intelligent tutoring systems, stealth assessment, autograders, recommender systems, dashboards, peer feedback platform, dynamic scaffolding, peer-to-peer student communication, Internet of Things (IoT)

We also solicited the views and ideas of subject matter experts and other stakeholders in STEM higher education, including those from traditional 4-year colleges and universities, online programs, and technical and community colleges.

In order to frame the examination of where and how the ILE category may impact the students’ experience, we address the following framing questions (FQ) related to the four ILE categories. In particular, we examined the questions from the context of impact on the various aspects of teaching and learning: content presentation, interactions and communications, learner activities, assessment, and co-curricular activities.

FQ1. What are the *opportunities for gains* in the adoption of the ILE category?

FQ2. What *challenges/barriers* exist that may inhibit the impact of the ILE category on the creation of ILEs by 2026?

FQ3. What *implementation strategies* may be used to advance the ILE category in order to realize the impact by 2026?

FQ4. What *research questions* remain to be addressed in order to optimize the impact of the ILE category?

Below are our working definitions of the four domains of teaching and learning:

*Content Presentation and Instruction*: The substance or content of a course refers to the information about the subject domain that will be conveyed to the learner. The breadth and depth of the course content are selected to meet the instructional needs of the learner in order to achieve the course objectives.

*Interactions* *and* *Communications*: In the teaching and learning context, interactions may be defined as the exchange of information between class participants. Interactions can be between one to many (teacher to class or student to students), one to one (teacher to individual student or student to student), or group-based (teacher to group or group to group).

Teaching, the act of guiding, illuminating, and explaining the course concepts, facts, and experiences to the learner, is principally a function of interactions and communications. Multiple methods are used to teach course content, including the delivery of lectures, readings, viewings, discussions, and hands-on activities. The media formats used to teach the course content are also varied and may include speech, text, video, audio, and hands-on exercises.

*Learner Activities*: The student activities are methods used to engage the learner with the course objectives. These engagement methods require the learner to apply, practice, and further seek mastery of the course domain. Methods may include assignments such as practice problems, responding to questions, developing or constructing output that represents an understanding of the course concepts. Methods may also include hands-on activities such as lab exercises and experiments, and field studies.

*Assessment*: Assessing learners, progress in reaching the defined course objectives can be measured as the material is being delivered (formative evaluation) or at critical milestone markers (summative evaluation). Assessment strategies are viewed as instructional in nature as they may reveal to both the teacher and the student areas of needed improvement towards achieving the expressed learning outcomes.

*Co-curricular Activities* : The learner interacts with the course materials in many ways, internally to the course offering and externally through a variety of methods such as cultural and civic events, educational programs, internship and job experiences and even student-spirit and athletic events. The student experience consists of a wide range of interactions that may consist of athletic, academic, social, and service dimensions. Providing the student with access to a range of these dimensions is a critical link in the overall quality of the student experience.

For each framing question, we consider how the ILE category impacts the presentation of content, the interactions and communications between and among learners and instructors, and the methods and techniques used to conduct assessment of student progress. These four framing questions, combined with the four ILE categories, create a 4x4x5 3D matrix shown in Table 2.

**Table 2:** 

	Personalized and adaptive learning	Multimodal learning formats	Cross reality	Artificial intelligence and machine learning
Opportunities for learning gains	Context-setting framework: • Content presentation • Interactions and communications • Learner activities • Assessment • Co-curricular activities			
Challenges and barriers				
Implementation strategies				
Research questions				

In the following chapters, we will present our in-depth analysis of each of these combinations.

## X-FILEs Workshop Activities

Drs. Ryoo, Ragan, and Winkelmann hosted three online meetings in the months preceding the 2018 workshop. These introduced the technology categories and solicited feedback for each framing question from participants working in small groups. The online discussions were structured to stimulate broad ideas and visions of the potential for ILEs impacting STEM higher education in 2026. Participants included education administrators, instructional designers, STEM researchers and faculty, and industry experts. Some attended the subsequent workshop, while others could only attend the online meetings. A total of 100 people attended the online meetings.

**Fig. 1:**
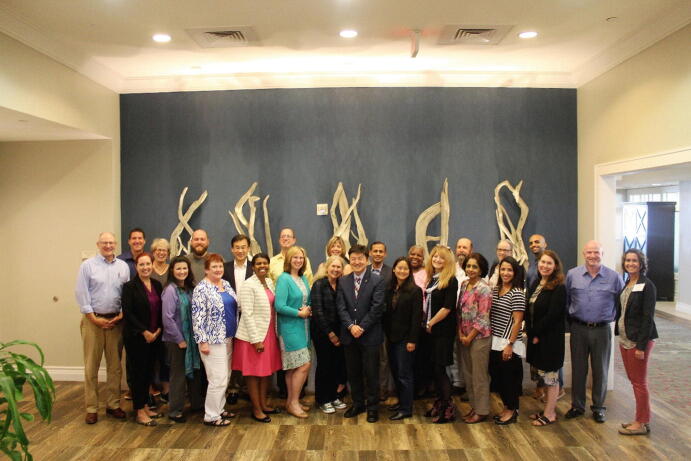
X-FILEs organizers and participants

Organizers prepared a list of potential workshop attendees based on members of their own professional networks, educators previously funded by NSF, online meeting attendees, and others who completed the online registration. Organizers selected a diverse group of attendees that included education researchers and practitioners in all four technology areas (Fig. 1). They adjusted the workshop’s agenda and structure based on comments, ideas, and feedback from the online meeting participants.

The 2-day, face-to-face workshop in Melbourne, Florida, provided attendees a chance to collaboratively engage in creative activities that helped them exchange ideas and views on the use of ILEs to transform higher STEM education learning. The workshop commenced with a welcome dinner and two keynote talks. Dr. Virginia Tickles spoke about her professional life as a NASA engineer and her experiences as a strong advocate and promoter of STEM education. In the second talk, NSF DUE Program Officer Dr. Pushpa Ramakrishna described the efforts of NSF to create a vision for the future of STEM education, to which this workshop was contributing.

Day 1 started with a “Lightning Strike” question session as an ice breaker activity. Resembling the World Café Method (Brown 2002), participants were encouraged to move between “question stations” that promoted discussions around the four ILE technology categories under study. Once this data was collected, a set of activities allowed for a deeper look of each technology category.

**Fig. 2:**
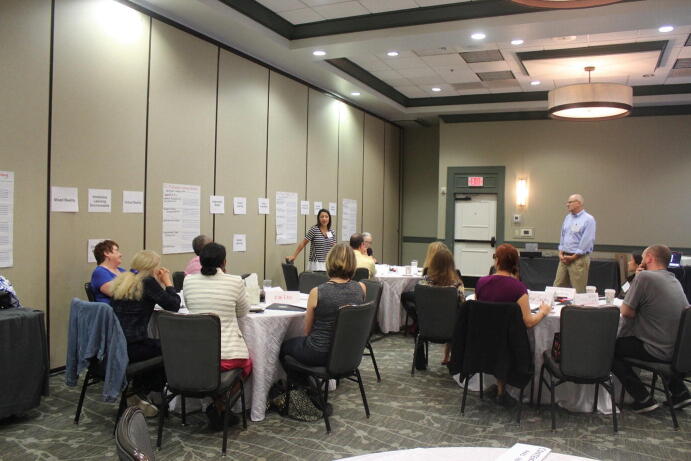
X-FILEs group activities

Dr. Larry Ragan, the workshop moderator, divided attendees into five teams, one for each of the aspects of teaching and learning. Participants in each team explored the framing questions related to personalized and adaptive learning. A team lead and recorder compiled the team’s thoughts and transcribed them in a pre-structured online document. At the conclusion of the discussion, all attendees reconvened and shared highlights of their discussions with each other. This same activity was repeated in the afternoon session for the second category, multimodal learning (Fig. 2).

Day 2 began with participants creating four narratives, each describing “A Day in the Life” of a higher education student in the year 2026. Stories were developed around one of these four assigned personas: (1) unconventional or alternative learner, (2) rural student attending a university fully remotely, (3) residential student, and (4) career-changing adult learner. These stories with “big ideas” were later shared, captured, and recorded.

To explore the last two technology categories, four posters for XR and four posters featuring AI and ML were created. Each contained one framing question along with a wheel depicting the five aspects of teaching and learning. This gallery walk exercise (Carleton College 2018) encouraged all workshop participants to post ideas, reactions, and considerations within each section of the wheel. Teams then distilled and organized the contents of these idea sheets in order to reveal trends and themes and reduce redundant posts. Using this data, teams organized and structured a response for each of the aspects of teaching and learning. Teams reported results to other participants to foster comments, modifications, and agreement.

This day culminated with a student-led discussion panel in order to gain insights into the student perspective of ILEs in STEM education in 2026. These four student panelists included a current high school junior, two undergraduate students, and a graduate student. Following a series of prepared questions, the workshop participants and panelists engaged in a conversation regarding the expectations and anticipations of the higher education experience in 2026.

Following the event, the workshop organizers invited participants to contribute to a white paper describing the workshop outcomes that later evolved into this book. When necessary, they invited other contributors not present at the workshop to help write and review chapters.

## ILEs Addressed by X-FILEs Project

Advances in educational technology must be coupled with a deep understanding of how students learn and retain information. Only then can developers create new, innovative ways for students to interact with their course materials that replace or augment traditional approaches inside and outside of the classroom. Although some of the ILEs discussed in this book are heavily dependent on technology, they are also part of sound pedagogical approaches that promote student-centered learning. Some major ILEs listed in Table 1 are briefly described below and may involve a physical space, a virtual space, or a combination of the two.

“ Digital storytelling is the modern expression of the ancient art of storytelling” (Barrett 2006) and has become increasingly popular in recent years, as shown well in the examples of YouTube and podcasting. Digital stories are often in the form of multimedia movies that incorporate photos, animations, videos, soundtracks, texts, and narrations. College students are comfortable using diverse platforms and tools (e.g., Unity, OpenSimulator, Xtranormal, or Garry’s Mod) to make, play, and share interactive digital stories (Pappas 2013). Many educators are already using digital storytelling in their classrooms to enhance the educational experience of their students. To be able to tell their own digital stories, students have to go through a rigorous process of researching a topic, developing a specific point of view and insights, and finally sharing them in creative ways. This experience is germane to one of the ultimate goals of higher education: producing independent learners.

The Internet of Things (IoT) is a growing field of digital communications that allows objects to communicate with each other, creating smart environments that respond appropriately using data collected by ubiquitous sensors. It is expected that the IoT will have a significant impact on many economic sectors (Manyika et al. 2015). The IoT can impact education in two ways: firstly, students can access more data from interconnected physical objects, either nearby or in a remote location. This data provides students with a richer, more detailed understanding of their environment, and they can use the data to study real-world challenges (i.e., through problem-based learning) (Ali et al. 2017). Secondly, sensors can monitor a student’s facial features and other characteristics. This data shows when a student is under stress or not fully engaged in the learning process (Farhan et al. 2018). A teacher could then call for a break or redirect the student’s attention back to the activity.

Micro-credentials and digital badges acknowledge accomplishments and skill acquisition at a more granular level than college degrees. Many corporations and online programs already adopt micro-credentialing and badging. IBM offers digital badges for its data science training through its IBM Skills Gateway (IBM 2018). Their digital badges recognize the qualifications of IBM’s own employees as well as the public in general. edX, a nonprofit online education consortium of higher education institutions, has introduced MicroMasters programs. Universities are also actively looking into ways to incorporate micro-credentialing and badging into their curriculum (Fain 2016). Some of the benefits include more explicit and organized guidance on students’ academic achievements and higher motivation and engagement.

Online learning , in the form of massive open online courses (MOOCs), has existed for many years, but MOOCs remain an innovative method for learning due to their continuous incorporation of new technologies (e.g., wikis, credentialing, and personalized learning algorithms). MOOCs hold great promise for democratizing education, but this ILE has not reached its full potential. Only a fraction of science and engineering subjects are offered by MOOCs. Less than 10% of students enrolled in a MOOC actually finish it (Lakshminarayanan and McBride 2015). Although MOOCs are available to groups who have been traditionally excluded from higher education, most users are employed, male, and live in countries of the developed world (Christensen et al. 2013). In a blended learning environment, the curriculum is presented in both an online format and in a traditional classroom space. For instance, students may complete the “lecture” portion of a chemistry class online but travel to campus to complete their laboratory experiments (Dalgarno et al. 2009).

Flipped classrooms are another example of blended learning. In a flipped classroom setting, students perform technology-rich exercises that prepare them for learner-centered activities during class. Pre-class activities can include videos, interactive computer-based simulations, and social media (Lundin et al. 2018). In-class activities require students to critically think about the subject matter and its applications (O’Flaherty et al. 2015). Flipped classrooms are growing in popularity, and they are used frequently to teach STEM subjects. Student acceptance varies; some enjoy their active role in the classroom, and others resist this format because it requires their greater preparation (McNally et al. 2017).

Game-based learning (GBL) leverages the entertaining experience of computer or noncomputer games to achieve an educational objective such as changing attitudes (Stewart et al. 2013) or improving knowledge in subjects such as math (Castellar et al. 2015) and physics (Hamari et al. 2016). Games may be designed with specific learning objectives in mind, or they can be developed independently (e.g., a commercially available video game with no particular educational goal) that nonetheless can lead to users achieving desired cognitive or affective learning outcomes. Purposefully designed educational games are known as serious games. Their use has been shown to improve student learning (Clark et al. 2016; Vlachopoulos and Makri 2017), and best practices for studying their effectiveness are available. However, more rigorous assessment methods are necessary in order to clearly understand how GBL benefits students (All et al. 2016).

Virtual environments positively impact students’ attitudes and learning (Hew and Cheung 2010; Kim and Baylor 2015; All et al. 2016; Winkelmann et al. 2017). Examples of virtual environments relevant to this book include virtual, augmented, and mixed realities and AI-enhanced virtual environments. Game-based learning environments may also have a virtual component.

Computer-based simulations and virtual lab experiments, such as PhET and Late Nite Labs, are effective supplements to many STEM courses (Moore et al. 2014; Pyatt and Sims 2012; Finkelstein et al. 2005; Reece and Butler 2017). Immersive virtual reality (VR) allows the user to experience virtual worlds more deeply by viewing the virtual world through stereoscopic visual displays within a headset. This blocks out other sensory distractions and provides a truly three-dimensional view of the virtual objects and other avatars within the VR program. Motion sensors track users’ head and hand motions in real time to create an immersive experience in which users control their avatars and interact with each other in the virtual reality setting. One goal for VR-based education is for students to experience the psychomotor “hands-on” aspect of a real-world lab experience. VR technology is used or is being developed for aviation (Koglbauer et al. 2011; Yin et al. 2014), medicine (Buckley et al. 2012), and training in other fields (Kinateder et al. 2015). Virtual reality will provide students with more opportunities for learning both in and out of the classroom (Ma et al. 2014; Lakshminarayanan and McBride 2015). Augmented reality (AR) allows a user to view and interact with virtual objects layered over real objects when viewed through a headset or mobile device screen. It is a similar technology to VR, and its implementation is generating beneficial outcomes for students (Cai et al. 2014; Borrel and Fourches 2017). In a mixed reality (MR) environment, the virtual content and the real content coexist, and the virtual content responds to changes in the real environment. Collectively, these ILEs are referred to as cross reality (XR). Activities can be collaborative and inquiry-based, depending on how the virtual world is designed.

The use of smartphones and tablets for education is known as mobile learning or m-learning (Lakshminarayanan and McBride 2015). These devices enable students to participate in many other ILEs. For instance, smartphones are useful for collecting and processing IoT data. They provide a display screen for augmented reality devices and run educational game apps. Since mobile devices are now ubiquitous among college students, it is reasonable to expect students to have access to assigned digital media which they view outside of class, as is done in a flipped classroom setting.

Digital publishing refers to the dissemination of digital content, such as text, videos, animations, and interactive visualizations, to the public. Digital publishing has many advantages over its conventional counterpart. For one, it can incorporate diverse digital media into publications. One of the emerging trends in digital publishing is open and independent digital publishing of learning knowledge. For example, version-controlled git-authoring (github.com) and open educational resources (OER) are emerging technologies in digital publishing (OER Commons 2018). Many believe that OERs are the future of educational content due to their more engaging, customizable, and affordable nature.

Intelligent virtual environments that integrate artificial intelligence techniques into the virtual environments may include XR and intelligent tutoring systems. For example, intelligent virtual reality systems (IVRS) incorporate AI algorithms into virtual reality (Aylett and Cavazza 2001). Advances in AI have allowed for the development of virtual environments that incorporate agents, eHealth-related devices, human actors, and emotions, projecting them virtually and managing the interaction between all the elements (Rincon et al. 2017). In these environments, the simulation and detection of human emotions can be used for the improvement of the decision-making processes of the developed entities. Machine learning occurs as the AI system adjusts its own algorithms in order to achieve greater success at its task. AI and ML have tremendous potential to enhance creative inquiry and informal learning, as well as adaptive learning through intelligent tutoring systems. One example is a web-based AI tutoring system in Tunisia that recognizes facial expressions as students progress through science experiments that they can access from anywhere (Adams Becker et al. 2016). Though most of the educational AI software is still in the development stages, advancing technologies could drastically alter the landscape of how students learn (Lynch 2016).

Stealth assessment is the evaluation of a learner’s performance in a manner that is unobtrusive, using metrics embedded in the course material (Shute and Kim 2014). Students learn without realizing that they are being constantly monitored for their progress. Another benefit is the immediacy of feedback. The educational content can adapt to how well students are doing at a given moment. Technologies now allow educators to conduct stealth assessment without the concerns of costs or system performance. Educators can closely examine the learning process of individual learners in a group by observing a student’s collaborations and contributions without disrupting the group.

## Education Technology Trends Identified Prior to the X-FILEs Project

A discussion of the future use of ILEs in STEM education brings to mind a cautionary Danish proverb: It is difficult to make predictions, especially about the future. Therefore, it is worthwhile to look back at past predictions in order to see how useful such predictions might be, which predictions were in fact credible, and how intervening events derailed expectations of other technologies.

A ready source of informed predictions is provided by the annual New Media Consortium (NMC)’s Horizon Report for the use of technology in higher education. In addition to identifying and analyzing challenges and trends in higher education (not just STEM education), the Horizon Report describes technologies that are poised for widespread adoption in the near, medium, and long term. The 2013 and 2014 Horizon reports (Johnson et al. 2013, 2014) predicted that 3D printing and wearable technologies in 2013 and quantified self and virtual assistants in 2014 were emerging technologies that will be widely adopted in higher education within 4–5 years (2017–2019, during the time period of the X-FILEs project).

3D printing is the design and fabrication of three-dimensional objects by depositing successive layers of the printing material. A user first creates or downloads a digital file that the printer uses to build the desired object. Ceramics, metals, polymer composites containing metal particles or wood fiber, and even paper are commercially available, but the most common printing materials are plastics (von Übel 2019). 3D printing is the most popular technology used in makerspaces—workshops where students and hobbyists collaborate, learn, and use low- and high-tech tools to create. Consumer-grade printers are inexpensive, and websites allow users to share designs. This technology and makerspaces in general are indeed becoming increasingly common in schools, as reflected by the 2018 NMC Horizon Report which placed widespread adoption of makerspaces within a year (Adams Becker et al. 2018). Students can use 3D printing in many STEM activities for chemistry (Pinger et al. 2019), engineering (Chien et al. 2018), and biology (Gordy et al. 2020). A recent review article (Ford and Minshall 2019) describes the current state of this technology in education. The two most common ways of incorporating 3D printing in STEM education are rapid, inexpensive production of models that improve student learning and teach the skills associated with designing the virtual 3D model (e.g., CAD) and operating the 3D printer.

Wearable technology (wearables) is a category of the Internet of Things (IoT) devices that can incorporate other technologies: smartwatches, exercise tracking devices, AR glasses, VR headsets, smart hearing devices (hearables) that interact with virtual assistants or translate speech, and a growing number of proposed medical sensors that track a wearer’s health conditions. Sensors within the wearable transmit data to a mobile computing device or to a remote cloud server for analysis and display for the wearer. This is desirable if the wearer wishes to improve quality of life and increase self-awareness. Real-time, passive, personal data collection is the basis for the quantified self-movement noted in the 2014 NMC Horizon Report.

Wearables highlighted in this report include AR glasses and VR headsets. These technologies have shown the most growth in education during the past 5 years. AR and VR in the consumer entertainment industry increased students’ acceptance, generated best practices for improving the student’s virtual experience, and popularized development tools for creating educational VR experiences.

Potential wearable applications associated with the quantified self have not advanced as rapidly due to a variety of limitations and challenges. These include the need for a continuous wireless connection to a user’s phone and the difficulty of displaying information on small screens (Kemper 2019). Medical sensors must first undergo rigorous study and review by healthcare regulatory bodies such as the US Food and Drug Administration (FDA). Collecting, transmitting, and analyzing a wearer’s personal health data raise privacy concerns as well. It is not clear how these wearables would be used in educational settings, although students could use such information to improve their diet and sleep schedule, which could impact their ability to learn (Johnson et al. 2014).

Virtual assistants are now well known, thanks to Hey Google, Apple’s Siri, and Amazon’s Alexa. A user speaks (or types) to the virtual assistant using conversational language, which interprets the words and context and then gathers relevant information from databases or other apps. Virtual assistants use machine learning to improve their understanding of the user’s speech and the context of questions. A particular brand of virtual assistant is designed to seamlessly interact with other technologies developed by the same company. These virtual assistants can answer questions about arithmetic, historical dates, and basic science facts. Some virtual assistants allow third-party development of add-on features that can provide more specialized information. A review of the research literature does not show significant implementation of commercially available virtual assistants in higher STEM education.

Conversational agents, more commonly known as chatbots, are another type of virtual assistants that are more widely used inside and outside of education. A chatbot answers questions or provides information about a specific topic, such as a company’s chatbot providing customers with frequently requested services. The most well-known example of this was Goel’s use of Jill Watson, a virtual teaching assistant designed to answer students’ questions about policies and assignments for his online AI course (McFarland 2016). This application has been employed by others as well (Chopra et al. 2016). Educators can teach a subject by employing a chatbot to conduct conversations with students within a virtual world (Heller et al. 2016). This is especially useful in medical schools to help students learn to converse with patients (Carrard et al. 2020). Virtual tutors can help students learn course content outside of class at their own pace (Coronell et al. 2019).

This brief and admittedly inexhaustive review of previous predictions about education technology use shows that many such predictions are accurate, even when made 5 years in advance. The most widely used technologies have commercial applications as well, such as 3D printing, AR and VR, and virtual assistants. Familiarity with technology can encourage its adoption by faculty and acceptance by students. Other factors that promote the use of new technologies are the ability to lower costs, as in the case of 3D printed models and virtual patients, and provide more personalized learning outside the classroom, such as virtual assistants that save time for faculty and increase convenience for students.

## Conclusion

This book describes:

- Current and future ILE development opportunities, including the potential for improving academic achievement and noncognitive outcomes (e.g., self-efficacy) among students
- Visions toward solutions to technological, logistical, administrative, and societal challenges inhibiting the realization of the potential of ILEs
- Implementation strategies that advance the potential impact of ILEs
- Research domains requiring additional exploration, analysis, testing, and reporting to maximize the impact of ILEs on STEM higher education by 2026

We hope that our book will help guide education policy-makers, researchers, developers, faculty, and practitioners to make informed decisions about the adoption and use of ILEs.

### Overall Impacts

Throughout our project, we take a holistic view of ILE implementation in higher education by considering not only the benefits of ILEs but also addressing the problems that they create and the challenges to their implementation. For instance, how can educators use technology-based ILEs without exacerbating the digital divide among students, and what policies (either within a school or at the state or federal level) must change in order to promote the adoption of ILEs? By thinking beyond the education research findings about ILEs, we are hoping that this book is creating a solid foundation for developing a greater understanding of how society and educational innovations can influence one another. These considerations lead to more challenging problems, but the solutions envisioned in this book will be more realistic and successful when implemented.

### Limitations

A challenge of integrating many of these ILEs into a curriculum is that the physical space is often set up for a traditional lecture or study area, but many academic activities require group work. This necessitates a rearrangement or remodeling of the classroom. Easily movable chairs, accessible electrical outlets for mobile devices, rearrangeable desk space, and whiteboards available for small groups enable students to engage in learning without the limitations imposed by a classroom design. Students value the functional attributes of a learning space and have definite opinions about where they can effectively study (Beckers 2016). Proponents of new education technology must appreciate how it fits into existing physical structures and campus cultures.

### Future of X-FILEs

The X-FILEs project has been evolving and will continue to adapt to the newly emerging challenges. As we finish our book, the world is in the middle of a global pandemic. COVID-19 caused college faculty to quickly change the way they teach traditionally hands-on STEM activities (e.g., lab experiments) to online instructional settings. This is an example of emergency remote teaching (ERT). Through a survey, online discussions, and interviews, the research team will document, curate, and study the ERT experiences of faculty who used innovative learning environments (ILEs) despite having little to no prior experience with them. Potential ILEs include synchronous and asynchronous teaching by video, use of virtual laboratory activities, and other emerging technologies. Selected faculty participants will have limited ILE teaching experience since this group teaches the majority of undergraduate STEM courses and is most in need of assistance implementing these technologies. It is expected that most participants will share the challenges that they faced; others may have discovered unexpected advantages of using ILEs. They will also convey how their experiences shape their attitudes toward future use of ILEs in STEM education.

This is what our focus is for now, but we are certain that our future projects will continue to take us to many other unexplored areas of the creative use of ILEs in addressing challenges in STEM education.
